# Enhancing mechanical properties of chitosan/PVA electrospun nanofibers: a comprehensive review

**DOI:** 10.3762/bjnano.16.22

**Published:** 2025-02-26

**Authors:** Nur Areisman Mohd Salleh, Amalina Muhammad Afifi, Fathiah Mohamed Zuki, Hanna Sofia SalehHudin

**Affiliations:** 1 Forest Products Department, Forest Research Institute Malaysia, 52109, Kepong, Malaysia; 2 Department of Mechanical Engineering, Faculty of Engineering, University of Malaya, 50603 Kuala Lumpur, Malaysia; 3 Department of Chemical Engineering, Faculty of Engineering, University of Malaya, 50603 Kuala Lumpur, Malaysia

**Keywords:** biomaterials, chitosan, electrospun nanofiber, mechanical properties, polyvinyl alcohol

## Abstract

This review examines strategies to enhance the mechanical properties of chitosan/polyvinyl alcohol (PVA) electrospun nanofibers, recognized for their biomedical and industrial applications. It begins by outlining the fundamental properties of chitosan and PVA, highlighting their compatibility and mechanical characteristics. The electrospinning process is discussed, focusing on how various parameters and post-treatment methods influence fiber formation and performance. Key strategies for improvement are analyzed, including material modifications through blending and structural modifications like fiber orientation and multilayer constructions, and surface modifications such as coating and functionalization. The review also covers advanced characterization methods to evaluate mechanical properties and provides a comparative analysis of different enhancement approaches. Applications in biomedical and industrial contexts are explored, showcasing the versatility and innovation potential of these nanofibers. Finally, current challenges are addressed, and future research directions are proposed to overcome these obstacles and further enhance the mechanical properties of chitosan/PVA electrospun nanofibers, guiding their development for practical applications.

## Introduction

**Figure 1:**
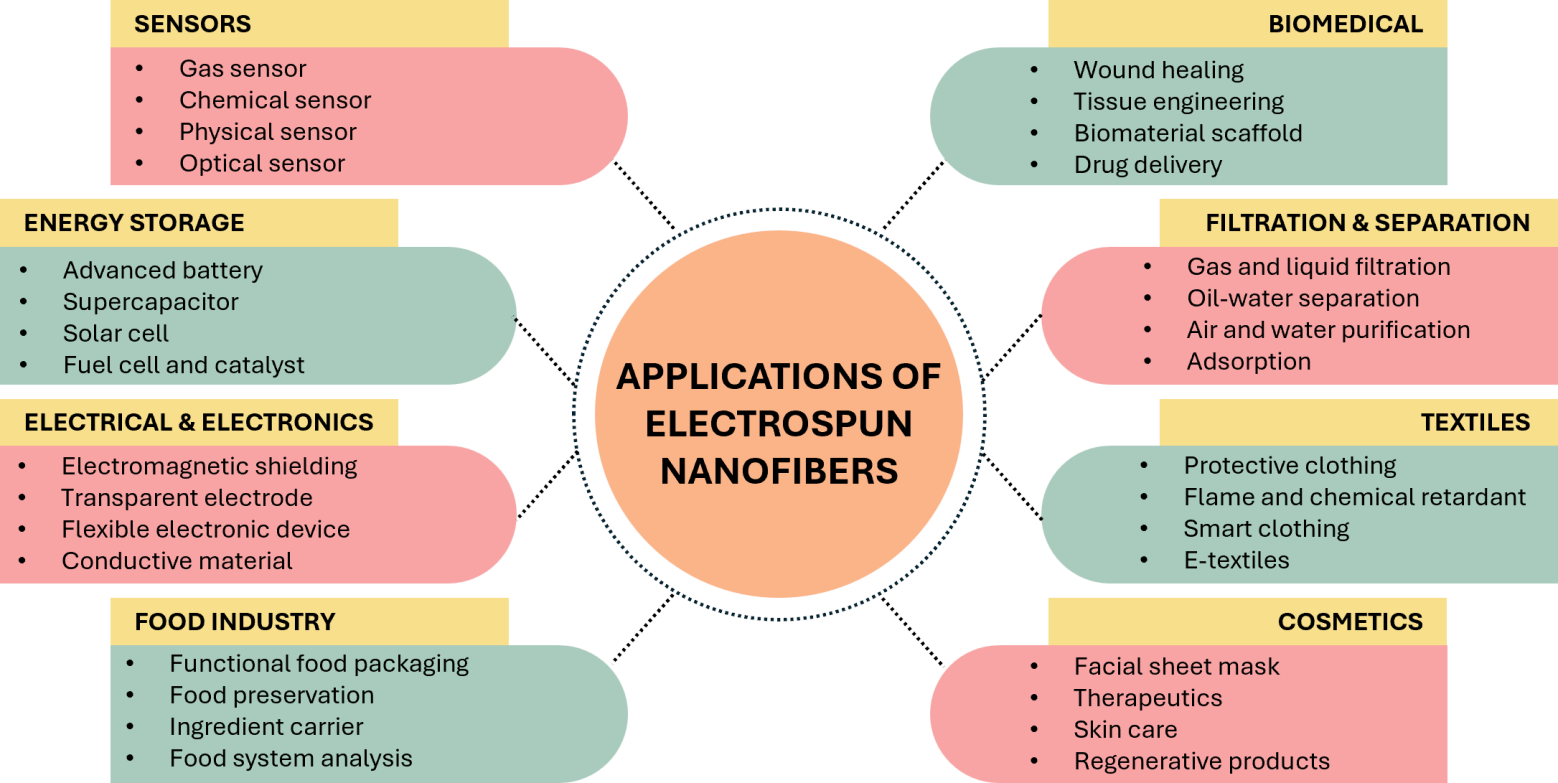
Applications of electrospun nanofibers [10–11].

In recent years, electrospinning has attracted significant attention from scientists because of its easy process [1]. Electrospinning can fabricate polymeric fibers ranging from the micro- to the nanoscale [2]. It is an easy, simple, and low-cost technique that does not require heat, an important factor for sensitive compounds [3]. Electrospun nanofibers exhibit a large surface area, high porosity, and small pore size, making them useful for a wide range of applications, as shown in Figure 1. Chitosan/polyvinyl alcohol (PVA) electrospun nanofibers have many applications, including water treatment, biomedical uses, and wound healing [4–6]. However, a drawback of electrospun nanofibers is their mechanical properties [7–8]. Electrospun nanofibers typically exhibit poor mechanical properties due to their high porosity, random fiber arrangement, and weak interactions at the cross-points of the nanofibers [9]. In this review, we focus on the background of the electrospinning process, the properties of chitosan/PVA electrospun nanofibers, and fabrication techniques, including the effects of various parameters and post-treatment methods. We also review the characterization of chitosan/PVA electrospun nanofibrous membranes and the methods to improve their mechanical properties, applications, and future perspectives.

## Review

### Background

Electrospinning is an advancement of electrospraying, where electric forces are used to disperse fine aerosols from a polymer solution, a technique invented in 1747 by Abbé Nollet [12]. During electrospinning, fluid is drawn through an electrically charged spinneret with a conical shape at the tip from which a jet emerges [13]. Based on Zeleny’s research, Geoffrey Taylor described in 1964 that the meniscus’ critical half-angle approaches 49.3° immediately before collapse. Thus, in a strong electric field, the tip of a polymer solution or melt extruded from a capillary changes from a spherical to a conical shape, the so-called Taylor cone [14].

The parameters involved in the electrospinning process can be divided into three categories, namely, electrospinning, solution, and environment. For electrospinning, factors such as applied electric field, distance between the needle and collector, flow rate, and needle diameter affect the fabrication of the nanofibrous sample. Solution parameters include the types of solvent, polymer concentration, viscosity, and solution conductivity. Finally, environmental parameters include relative humidity and temperature [15].

Chitosan, a widely utilized material in electrospun nanofiber membranes, is derived from the crystalline microfibrils of crustaceans, including crabs and prawns. It is biodegradable and exhibits a high capacity for adsorbing heavy metals and radionuclides [16]. However, chitosan exhibits limited mechanical stability, is sensitive to pH variations, and tends to swell [17]. To improve the spinnability of chitosan during the electrospinning process, it is commonly blended with other polymers, such as PVA [18]. Additionally, PVA contributes to reducing the crystallinity of the chitosan structure [19].

Because of their unique and exceptional properties, nanofibrous membranes have become prominent materials for a wide range of applications [20]. Throughout their time of use, electrospun fibers are exposed to environmental stresses in each of these applications. These forces could cause the nanofibers to fail or become permanently deformed, potentially rendering the entire device inoperative. Thus, before using these materials in particular fields, it is essential to take their mechanical properties into consideration [21].

Electrospun nanofibrous membranes typically have poor mechanical characteristics. The materials used, the non-Newtonian fluids in the electrospinning solutions, and the nanofibers’ random orientation are the three primary factors of the poor mechanical performance of electrospun nanofibers. For example, collagen is one substance used in electrospinning that has low strength [22]. Its loose structure and weak composition are the reasons behind its inherent poor mechanical performance. Electrospinning solutions with non-Newtonian fluids come next. They exhibit unstable properties, including irregular fluid jet movement, variations in surface tension, and sensitivity to external parameters such as temperature, humidity, and airflow. These factors can disrupt the uniformity of the fiber formation. When the fiber jets are drawn in the direction of the electric field force, the polymer chains in the fibers are not properly aligned, resulting in non-uniform mechanical and structural properties [23]. The incomplete crystallization of the fibers caused by the short and weak stretch process gives the fibers poor mechanical properties [24]. Last, there is the fibers’ random orientation. When an external force is applied to electrospun nanofibers, the load is distributed across a network of thousands of fibers. However, because of their random orientation, the force is not evenly distributed. In some cases, the load is concentrated on a single fiber, which can result in significant stress on that fiber and weaken the overall structure [25].

With advancements in technology for producing nanofibers through electrospinning, various methods and techniques have been developed to enhance the mechanical properties of electrospun nanofibers. The objective of this review is to explore current techniques and methods for improving the mechanical properties of electrospun nanofibers, thereby enhancing their application in various fields.

### Materials composition

**Figure 2:**
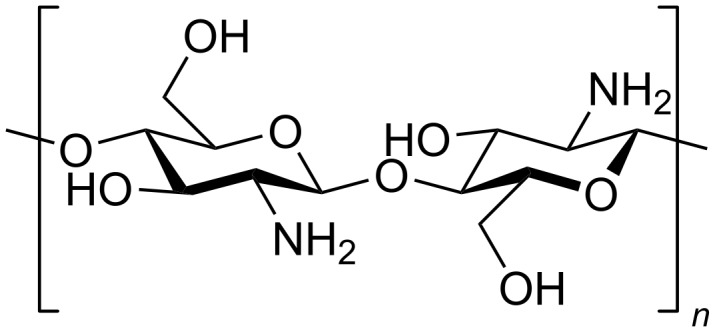
Chemical structure of chitosan.

Chitosan is an intriguing material derived from chitin. Because of its promising properties, it has attracted significant attention from researchers [26]. It is abundantly found in the shells of crustaceans and is the most abundant biopolymer after cellulose [27]. Figure 2 shows the chemical structure of chitosan.

Chitosan has been studied extensively and is recognized as a prominent material in the fields of medicine, food, and water treatment. Table 1 shows the properties applications of chitosan that have been studied.

**Table 1:** Properties of chitosan studied in various applications.

Industry	Property/application	References

medical health care	wound healing	[28–30]
	anti-oxidant	[31–33]
	antimicrobial	[34–36]
	antifungal	[37–39]
water treatment	nanofiltration	[40–42]
	chelating heavy metal ion	[43–45]
	removal toxic chemical	[46–47]
food	membrane as food packaging	[48–50]

Among the crucial factors in the electrospinning process is the solubility of the polymer [51]. Chitosan is soluble in organic acids such as diluted aqueous acetic, formic, and lactic acids, but it is insoluble in water, alkali, and most mineral acid systems. Additionally, chitosan is soluble in mixtures of water, methanol, ethanol, and acetone [52], making it feasible for the electrospinning process. Unbound amino groups make chitosan a positively charged polyelectrolyte in acidic media, resulting in high solubility [53]. This phenomenon makes it difficult to electrospin chitosan because of its high viscosity [54]. Moreover, the formation of strong hydrogen bonds in a 3D network contributes to difficulties in the mobility of polymeric chains when an electric field is applied [55]. Because of these factors, chitosan can only be electrospun in the presence of a spinnable polymer such as PVA [56]. A previous study has demonstrated the viability of adding PVA as an additional component to chitosan [57]. This addition reduces the repulsive interactions between polycations, thereby decreasing chain entanglement and promoting fiber formation [58]. A summary of the applications of chitosan/PVA nanofibers is provided in Table 2.

**Table 2:** Applications of chitosan/PVA electrospun nanofibers.

Sample	Applications	References

nanoclay-reinforced electrospun chitosan/PVA nanocomposite nanofibers	biomedical	[59]
chitosan/polyvinylpyrrolidone/PVA electrospun nanofiber	removal of heavy metal ions and organic pollutants	[60]
electrospun nanofibers of chitosan/polyvinyl alcohol/UiO-66/ nanodiamond	adsorbents for wastewater remediation and organic dye removal	[4]
electrospun chitosan/poly(vinyl alcohol)/glycerol nanofibers	skin care	[61]
biaxial electrospun nanofibers based on chitosan-poly (vinyl alcohol) and poly (ε-caprolactone) modified with CeAlO_3_ nanoparticles	wound dressing materials	[62]
electrospun chitosan-polyvinyl alcohol nanofiber dressings loaded with bioactive ursolic acid	diabetic wound healing	[63]
antioxidant peptide-loaded electrospun chitosan/poly(vinyl alcohol) nanofibrous mat	food packaging	[64]

**Figure 3:**
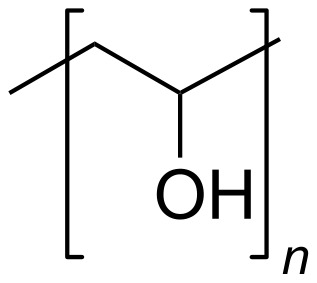
Chemical structure of PVA.

PVA is a synthetic polymer widely utilized in various industries because of its versatile properties. Its water solubility allows for a wide range of applications, while its carbon atom backbone ensures chemical stability and compatibility with diverse environments. Figure 3 shows the chemical structure of PVA. PVA is known for its biodegradability, allowing it to disintegrate in both aerobic and anaerobic environments. This feature is crucial for industries concerned about the environment because it mitigates the long-term effects of PVA waste on ecosystems. Research on PVA biodegradation further supports its status as an environmentally friendly polymer [65].

The mechanical properties of PVA depend on several factors, such as molecular weight and retained moisture [66]. Dry, fully hydrolyzed PVA exhibits ductile properties with an elongation at break of 25% [67]. However, PVA has low mechanical strength under wet conditions. Because of its solubility in water, its applications are limited, and it can only be used as an internal layer in multilayer structures. A convenient strategy to overcome these limitations, without losing the advantages of PVA such as biodegradability, is to blend PVA with stiff and water-insoluble biodegradable polymers such as chitosan [68].

### Fabrication techniques

**Figure 4:**
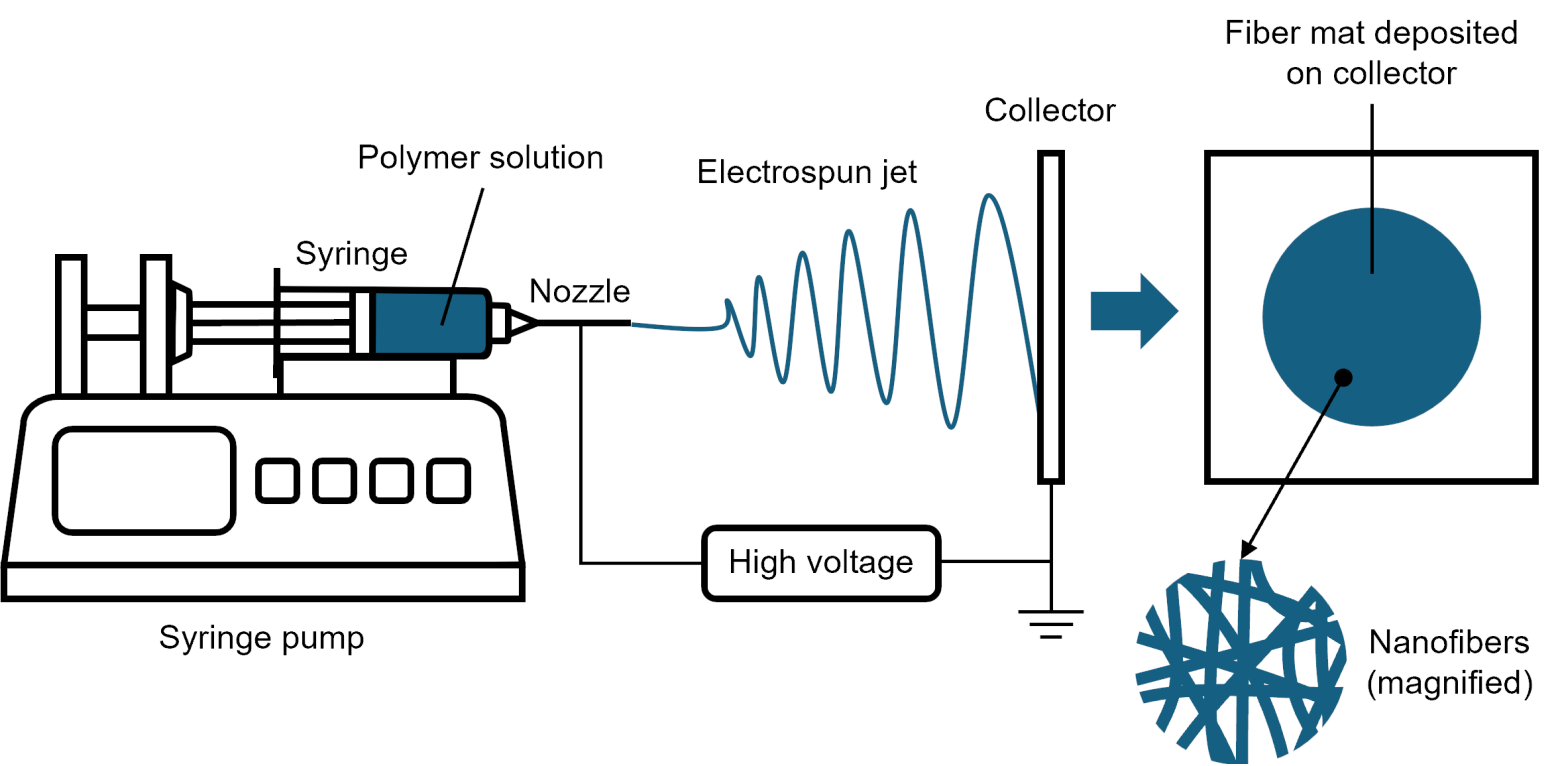
Schematic diagram of the electrospinning process for the fabrication of nanofibers.

Nanofibers can be fabricated using different methods such as direct drawing, template synthesis, phase separation, self-assembly, and electrospinning. Electrospinning is considered the most versatile and widely used technique for nanofiber fabrication, offering greater control over fiber structures, higher productivity, simplicity, lower cost, and potential for industrial use [69–70]. Electrospinning involves applying a high voltage to a polymer solution delivered through a nozzle or capillary [71]. The electrostatic charge builds up at the tip of the droplet, and when the repulsive force of the charges overcomes the droplet’s surface tension, a cone-like jet, known as the Taylor cone, forms, and the resultant solidified fibers are collected on a grounded (or oppositely charged) collector. The collector used can be stationary or moving, with different configurations affecting the alignment of the deposited fibers. A typical electrospinning setup configuration is shown in Figure 4.

Electrospinning is a versatile technique for producing microfibers and nanofibers, but the process, especially with the conventional single-nozzle electrospinning, has several limitations. Some of the challenges include low throughput, which hinders industrial-scale production, poor fiber uniformity, and limited control over fiber alignment and orientation [72]. Random fiber alignment and orientation, in addition to low crystallinity of electrospun structures, tend to result in poor mechanical strength [73]. To address the challenges of conventional electrospinning, researchers have explored alternative approaches with distinct fiber-producing capabilities such as centrifugal electrospinning, alternating current electrospinning, near-field electrospinning, multinozzle electrospinning, and needleless electrospinning. Other improvement efforts include modifications on the solution or melts used for electrospinning, innovative designs of the electrospinning setup and components, and various post-treatment methods. These advancements aim to improve productivity, safety, and control over fiber properties, potentially enabling broader industrial applications of nanofibers [12].

The use of a single polymer material as the electrospinning solution produces fibers with limited functional properties [74]. This limits the practical use of the fibers, especially to solve advanced issues that arise in line with the rapid growth in research and advanced technologies. Multicomponent fibers, composed of two or more materials, are designed to enhance the functionality and properties of the fibers, thereby broadening their applications in areas such as antibacterial treatments, water purification, and biomedical engineering [75–77]. Fabrication of multicomponent polymer nanofibers like chitosan/PVA can be carried out through methods such as blend electrospinning, sequential electrospinning, co-electrospinning, and emulsion electrospinning.

#### Blend electrospinning

**Figure 5:**
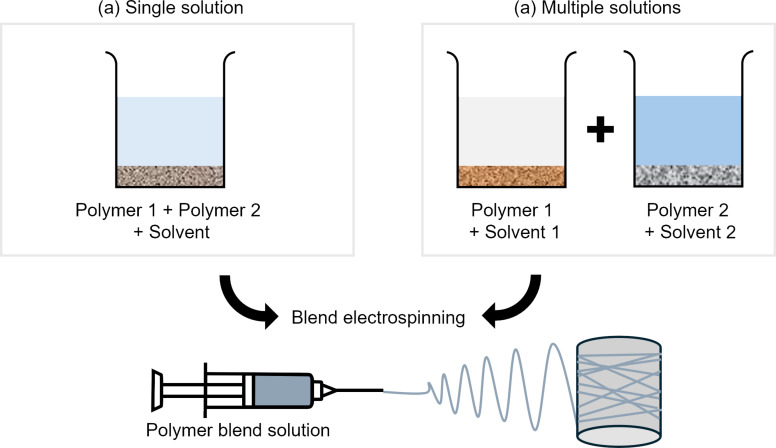
Electrospinning polymer blend solutions by (a) mixing two polymer materials in a single solvent system and (b) mixing two solutions prepared from separate solvent systems.

Blend electrospinning refers to the electrospinning of a single solution or mixture containing more than one material or polymer, which makes up one single-phase homogeneous liquid. Polymer blending allows for the creation of novel materials through the incorporation of the unique properties of the component polymers [78]. In blend electrospinning, the preparation of a homogenous solution is crucial in ensuring the formation of uniform fibers during electrospinning, and some of the important parameters to take into consideration are the molecular weight of each component, solubility of the solvents, temperature, and the polymer blend ratio [79]. The straightforward approach in preparing the solution is by directly mixing both polymers in the solvent system, which can be either a single solvent [57] or a mixture of solvents [80–81] (Figure 5a). The polymers can also be prepared separately in separate solvent systems [82–83], and the solutions are subsequently mixed to form the final solution before electrospinning (Figure 5b). The latter involves more steps but minimizes the risk of incomplete dissolution or phase separation due to different solubility requirements of each polymer [84]. Nevertheless, the instabilities can still occur when blending the two solutions if the solvents are incompatible.

#### Sequential electrospinning

**Figure 6:**
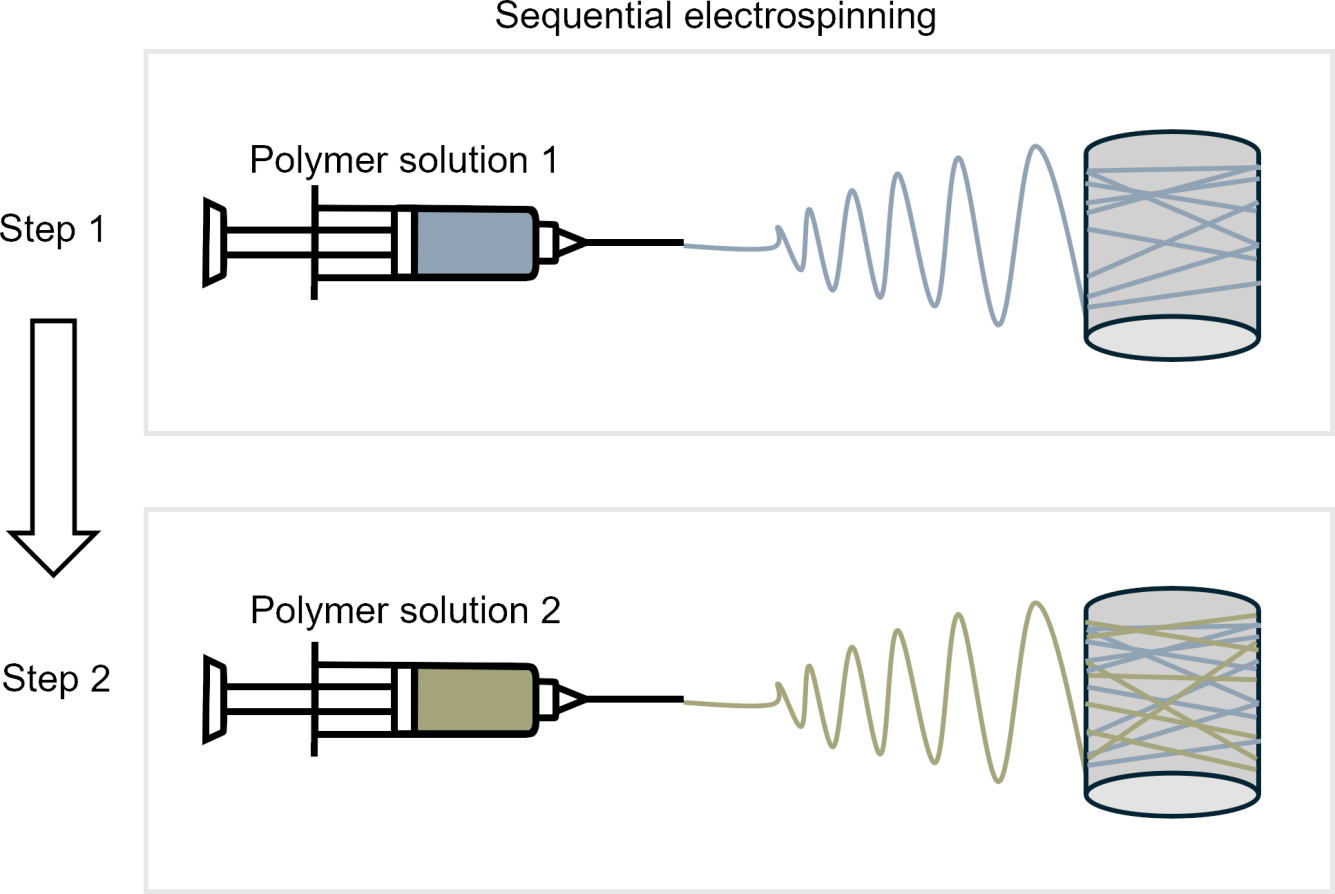
Two-step sequential electrospinning for the fabrication of multilayered nanofibers.

Sequential electrospinning is a method where two or more polymer solutions are electrospun consecutively to form a hierarchically ordered structure consisting of meshes of different materials [85]. Also called layer-by-layer electrospinning, the general steps of preparing multilayered composites using this method involve layering fibers upon fibers through multiple steps, as shown in Figure 6. The functionality of the overall structure depends on the different properties exhibited by each layer. In bioactive encapsulation and controlled release applications, for example, multilayered membranes can be used to regulate the release time of substances by exploiting the surface properties and interactions between hydrophobic and hydrophilic layers [86]. This sequential method of producing composite fibers is generally easier than the other methods as the different materials are electrospun separately, eliminating concerns related to compatibility of the materials and solvents during preparation as well as the interaction between the fiber jets during the electrospinning process. However, the entire procedure can become more time- and labor-consuming as more steps are required.

#### Co-electrospinning

**Figure 7:**
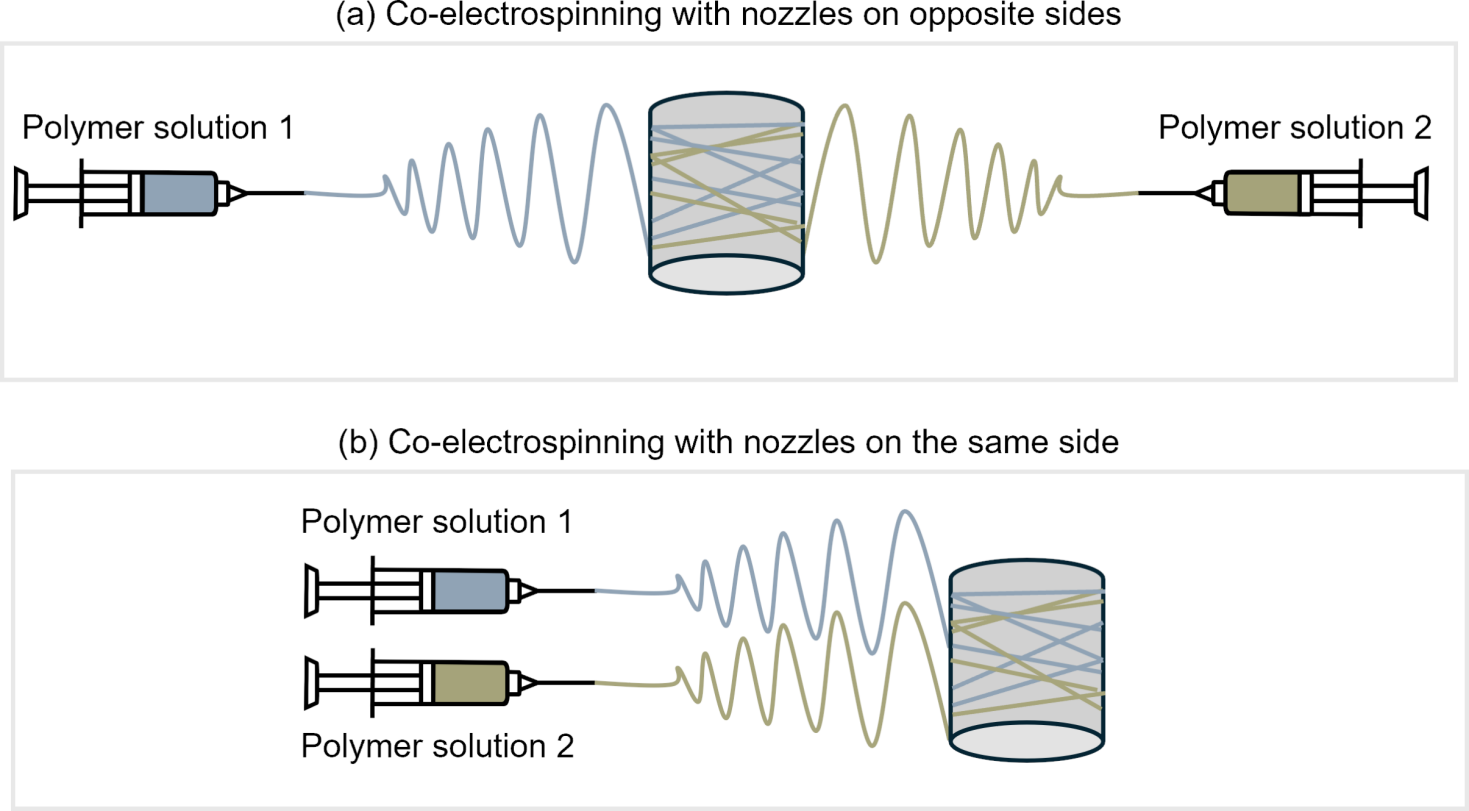
Co-electrospinning with two nozzles (a) facing each other on the opposite sides and (b) next to each other on the same side of the collector.

Co-electrospinning refers to the electrospinning of multiple solutions from multiple nozzles simultaneously (also known as multinozzle or simultaneous electrospinning). Like sequential electrospinning, multiple polymer solutions are prepared separately, but instead of layering one after another, the different materials are electrospun simultaneously, as shown in Figure 7. This method of electrospinning is usually preceded by single-nozzle electrospinning of each component separately to find the optimum parameters before running them concurrently [87]. Besides combining different fibrous materials, this multinozzle method can also be used to incorporate nanoparticles into the nanofiber structures through simultaneous electrospinning and electrospraying [88]. In co-electrospinning, the interaction between charged jets for nozzles that are positioned close to one another may cause jet repulsion for nozzles of the same polarity, which can cause difficulties in collecting the fibers. Nozzles that are placed on opposite sides as shown in Figure 6a can overcome this problem, but it requires a larger working area, and the use of rotating collectors is usually necessary. Other approaches include using oppositely charged nozzles so that the fibers attract instead of repelling each other, or using auxiliary electrodes to control the electric field and concentrate the flow of fiber jets into a smaller deposition area [89].

#### Multichamber electrospinning

Multichamber electrospinning incorporates special nozzle designs to fabricate multicomponent fibers, where a single nozzle is partitioned into separate chambers, with the most popular technique being the coaxial electrospinning. Coaxial electrospinning involves the use of a compound coannular nozzle to fabricate core–shell fibers [90–92]. For a two-solution system, the solution from the inner tube forms the core and the annular solution from the outer tube forms the shell of the resultant fibers. Although it appears as if only a single nozzle is used, the method essentially involves simultaneous electrospinning from multiple coaxial nozzles of different diameters; hence, it is sometimes considered a subcategory of co-electrospinning. The special design of the coaxial electrospinning spinneret enables fiber formation from polymer solutions that are considered almost impossible to electrospin on their own [93]. In this case, the shell fluid, which is usually the solution with better electrospinning capability, will assist in the entrainment of the core fluid to form core–shell fiber jets. Three-material core–sheath fibers are reported to have been successfully electrospun using triaxial nozzles [94]. Other design variants include having multiple nozzles enclosed within one outer nozzle, producing fibers with multiple cores encapsulated by a sheath material [95].

**Figure 8:**
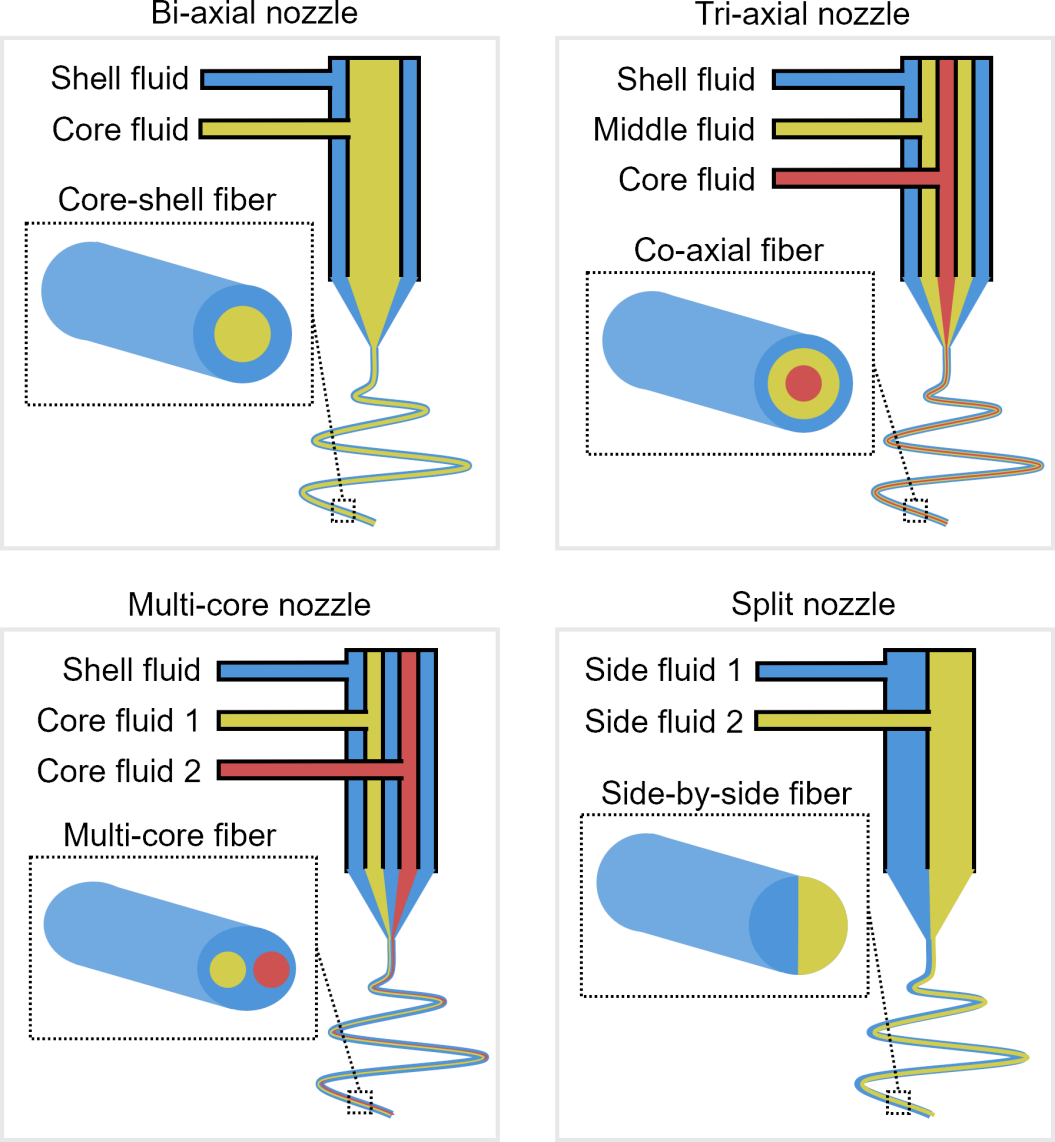
Multichamber nozzles used to prepare multicomponent electrospun fibers.

Another type of electrospinning based on a specialized spinneret is split-nozzle electrospinning, also referred to as side-by-side electrospinning, where the nozzle contains a wall or partition along the central axis, which separates two different solutions into two separate chambers within the nozzle [96]. The two different solutions should possess similar electrical conductivity for the materials to eject as a single jet without any phase separation. This technique can be regarded as a variant of coaxial electrospinning in which spinneret is conceptualized as two half-cylindrical nozzles joined together. The fibers formed from the process are attached side-by-side instead of a core–shell structure. Figure 8 illustrates the different types of fibers formed using multichamber nozzles.

#### Emulsion electrospinning

Emulsion electrospinning is similar to blend electrospinning in principle, but instead of a single homogenous mixture, the emulsion is based on multiple phases that are not mixed during the process [97–98]. To prepare an emulsion, two immiscible fluids, for example, oil and water are mixed with the use of stabilizers [99]. Emulsion electrospinning is a potentially sustainable method of electrospinning as it limits the use of organic solvents. This method also allows for the fabrication of core–shell fibers from a standard nozzle, without the need of coaxial nozzles. Emulsion electrospinning is a promising method for fabricating nanofibers with advantages such as protection, controlled release, and high loading efficiency for food, pharmaceutical, and biomedical applications [100].

Table 3 summarizes some of the main advantages and disadvantages of the different electrospinning methods employed to fabricate multicomponent nanofibers. The choice of method to use depends on the specific application and requirements of the fiber structure, considering the trade-offs between different techniques.

**Table 3:** Advantages and disadvantages of different electrospinning methods for multicomponent fiber fabrication.

Fabrication technique	Advantages	Disadvantages

blend electrospinning	simple setup and process as only a single nozzle is required; allows for mixing of multiple polymers in each fiber strand	difficulty in preparing stable homogeneous solution; limited control over individual polymer distribution within fibers
sequential electrospinning	ease of electrospinning without considering the interaction with the other material/solution; ability to form multilayer fibrous structure	possibility of weak layer adhesion; may be time-consuming because of discontinuity of the process
simultaneous multinozzle electrospinning	potentially time-saving because of simultaneous fiber production, ability to produce hybrid fiber mats and yarns; good fiber adhesion due to interwoven fibers	complex setup requiring multiple nozzles; possibility of processing issues and non-uniform fiber distribution due to electric field and jet interaction
multichamber electrospinning	ability to create distinct fiber morphologies such as side-by-side, core–shell, and encapsulated structures; saves space as the nozzles are partitioned internally	requires specially designed nozzles; difficulty to control the cross-sectional distribution of the multicomponent fibers; possibility of forming only a single-material fiber because of suppressed core fluid entrainment; risk of phase separation at the interface of the components
emulsion electrospinning	ability to create core–shell; encapsulated structures, or multiple phases without special nozzles; simple setup and process as only a single nozzle is required; potentially sustainable process by removing the need of organic solvents	difficulty in forming stable emulsions; risk of droplet coalescence leading to inhomogeneous fiber structures

### Fabrication of chitosan/PVA fibers

Chitosan nanofibers offer numerous advantages, including biocompatibility, biodegradability, and similarity to the extracellular matrix (ECM) [101]. Electrospinning pure chitosan without any other precursor material, however, presents significant challenges due to its inherent properties. Chitosan has high viscosity and low solubility in most solvents, and it tends to form gels, which complicates the electrospinning process. Repulsive forces between ionic groups due to high electric fields during electrospinning also tend to form discontinuous chitosan fibers [102]. Solvents used in the electrospinning of pure chitosan include hydrochloric acid, acetic acid, formic acid, hexafluoro isopropanol (HFIP), trifluoroacetic acid (TFA), and dichloromethane (DCM). TFA is a good solvent to produce homogenous fibers, but it is a highly toxic organic substance [103]. Because of the difficulties regarding the processing of chitosan, it is almost always mixed with another material prior to electrospinning, with polyethylene oxide (PEO) and PVA as popular precursor material candidates. Other disadvantages of chitosan nanofibers include low mechanical strength and poor solubility in water [104].

PVA is a good precursor material in electrospinning as it helps in improving the spinnability of fibers by reducing repulsive forces within charged polymer solutions [105]. While PVA nanofibers offer advantages such as ease of fabrication and biocompatibility, applications of single-material PVA nanofibers are usually limited by their poor mechanical properties, high water solubility and hydrophilicity, and low thermal stability [106–107]. The disadvantages often necessitate the combination of PVA with other materials or the application of post-processing techniques such as cross-linking or coating for performance improvement [108]. As a countermeasure, Rafieian et al. [109] proposed electrospinning only PVA, and preparing a separate film containing aloe vera and chitosan using a film applicator. The electrospun PVA was then layered on top of the aloe vera/chitosan film to improve mechanical properties. The chitosan/aloe vera film fabricated using this method, however, is in the form of a non-fibrous thin film; hence. it may not suit applications that highly depend on the properties of a nanofibrous structure, such as large surface area-to-volume ratio or high permeability of the material. Similarly, Kang et al. [110] fabricated chitosan-coated PVA nanofibers by coating the heat-treated PVA nanofibrous matrix with a chitosan solution to create a biomimetic nanofibrous wound dressing.

Electrospinning of chitosan/PVA blend nanofibers has been successfully reported by many researchers. In these studies, two of the most common solvents used to dissolve chitosan and PVA are water and acetic acid [82–83,103]. Thien et al. prepared chitosan and PVA solutions separately, dissolving chitosan in acetic acid and PVA in water. The two solutions were then subsequently mixed to form a homogenous solution for electrospinning [103]. The method of mixing the two solutions can also be adopted if the same solvent is used for the two materials, as reported by [82] and [83] with acetic acid or water as the only solvent, respectively. Vu et al. [57] prepared a solution by directly mixing chitosan and different concentrations of PVA in a single solvent system of acetic acid/water.

The use of multiple nozzles to fabricate chitosan/PVA nanofibers are also reported, but these studies involved electrospinning of chitosan and PVA together as a single blend from one nozzle, while the other nozzle contained different materials such as polycaprolactone (PCL) [62]. There are currently no documented studies reporting the simultaneous electrospinning of chitosan and PVA from separate nozzles, likely because of the good compatibility of these two materials in forming blends. Co-electrospinning of chitosan and PVA may complicate the process, since it is difficult to electrospin chitosan on its own. There have been reports of co-axial electrospinning of chitosan/PVA, although additional materials are usually added to aid the electrospinning of chitosan. Zhu et al. [111] used a chitosan/PCL blend as the sheath material and PVA as the core material for the production of guided bone generation membranes. Kuo et al. [112] added small amounts of gum arabic to significantly reduce the viscosity of the chitosan shell solution and to ease the electrospinning process while maintaining high chitosan contents. Interestingly, Chen et al. [113] fabricated trilayered fiber membranes via the combination of sequential, multinozzle, and blend electrospinning. The resultant composite was made up of chitosan fibers as the bottom layer, co-electropun chitosan and PVA fibers as the middle layer, and blended PVA/nanobioglass fibers as the top layer.

Emulsion electrospinning of chitosan/PVA was reported by Mouro’s research group in two studies [114–115]. The gel-like suspensions were prepared by mixing the aqueous chitosan/PVA solution and oil phase solutions including PCL and poly-ʟ-lactic acid for the use in wound healing applications. Instead of using nozzle-based methods, the emulsions were electrospun using a needleless technique based on a rotating cylinder spinneret to increase production rate. Table 4 summarizes the different electrospinning methods used in fabricating chitosan/PVA nanofibers.

**Table 4:** Methods and solvents for electrospinning of chitosan/PVA nanofibers from the literature.

Fabrication technique	Solution preparation	References

blend electrospinning (single solvent)	chitosan/PVA in acetic acid/water	[57]
	chitosan/PVA in plasma-acid	[80]
	chitosan/PVA/HA in acetic acid	[81]
blend electrospinning (multi-solvent)	chitosan in acetic acid + PVA in acetic acid	[82]
	chitosan in plasma-acid + PVA in water	[116]
	chitosan in acetic acid + PVA in water	[103]
	chitosan in water + PVA in water	[83]
	chitosan/TiO_2_ in acetic acid + PVA in water	[117]
	chitosan in acetic acid + PVA in water + alginate in glycerol	[118]
	chitosan in acetic acid/water + PVA in water	[119]
	chitosan in TFA/DCM + PVA in water	[120]
sequential electrospinning	layer 1: eudragit RL100 in methanol; layer 2: chitosan in acetic acid + PVA in water + ofloxacin; layer 3: eudragit RL100 in methanol	[121]
	layer 1: chitosan in TFA/DCM; layer 2: chitosan in TFA/DCM (nozzle 1) + PVA in water (nozzle 2); layer 3: PVA in water + nanobioglass	[122]
	layer 1: chitosan in acetic acid + PVA in water + AgNO_3_; layer 2: PVP or PEO in water + chlorhexidine	[123]
co-electrospinning (simultaneous multinozzle)	nozzle 1: chitosan in acetic acid + PVA in water + cephalexin + CeAlO_3_ nanoparticles; nozzle 2: PCL in acetic acid	[62]
	nozzles 1 and 2: keratin in water/NaOH + PVA in water; nozzles 3 and 4: chitosan in acetic acid + PVA in water	[124]
	nozzle 1: chitosan in acetic acid + PVA in water; nozzle 2: silk in HFIP	[125]
coaxial electrospinning	shell: chitosan/PVP in acetic acid + PVA in water; core: PEO in water (removed post-electrospinning to form hollow fibers)	[126]
	shell: PVA in water; core: chitosan/PCL in acetone/DMF	[111]
	shell: chitosan in acetic acid + gum arabic; core: PVA in water	[112]
	shell: chitosan in acetic acid + PVA in water; core: ascorbic acid in ethanol/propylene glycol/water	[127]
emulsion electrospinning	water phase: carboxymethyl chitosan/PVA in water + zinc oxide/argentum; oil phase: tea tree oil	[128]
	water phase: chitosan in acetic acid + PVA in water + lecithin; oil phase: peppermint essential oil	[129]
	water phase: chitosan in acetic acid + PVA in water; oil phase: PCL in chloroform/DMF	[115]
	water phase: carboxymethyl chitosan/PVA in water + β-cyclodextrin; oil phase: citral	[130]
	water phase: chitosan in acetic acid + PVA in water; oil phase: PCL in chloroform/DMF	[114]

### Characterization of mechanical properties

Electrospun nanofibers possess unique mechanical properties, which depend on their material composition, fiber diameter, and structural arrangement. The mechanical properties of PVA/chitosan-based nanofibers, such as tensile strength, elasticity, and elongation at break, are crucial for determining their suitability for various applications.

#### Tensile properties

**Figure 9:**
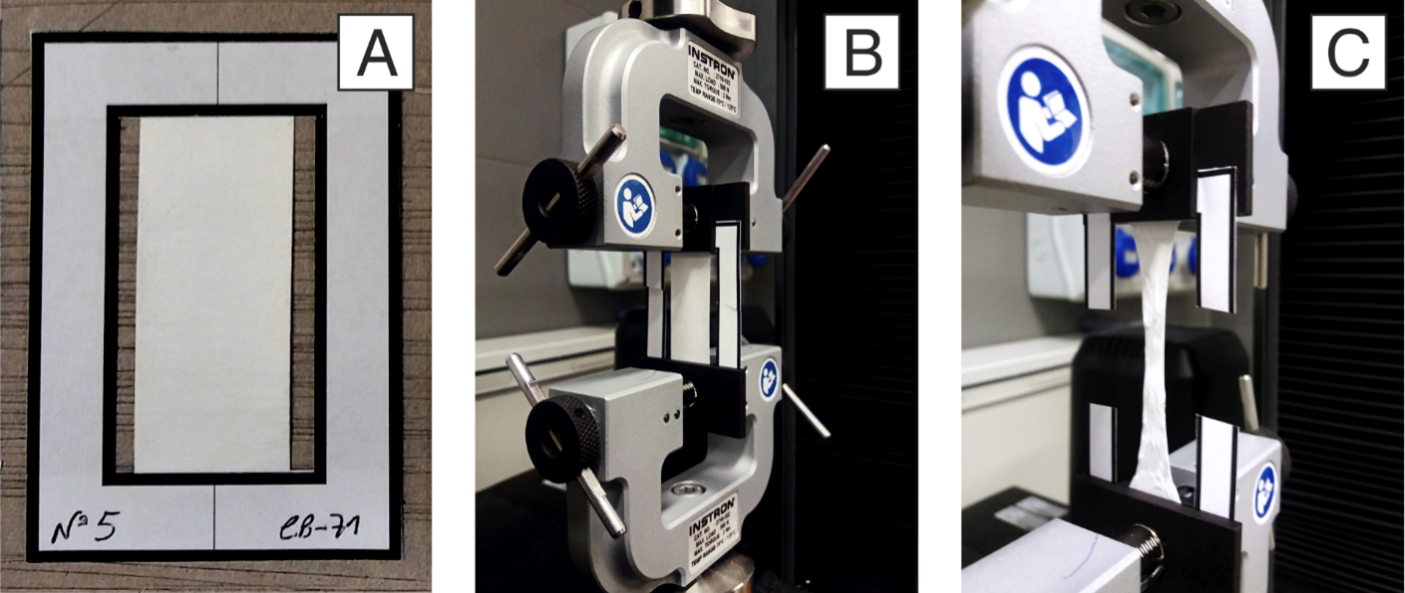
(a) Paper frame with test specimen, tesile test (b) before start and (c) during test. Reprinted from [133] Composites Part B: Engineering, vol. 166, by E. Maccaferri; L. Mazzocchetti; T. Benelli; A. Zucchelli; L. Giorgini, “Morphology, thermal, mechanical properties and ageing of Nylon 6,6/graphene nanofibers as Nano^2^ materials”, p. 120-129, Copyright (2019), with permission from Elsevier. This content is not subject to CC BY 4.0.

Tensile strength measures the maximum stress a material can withstand while being stretched before breaking and is one of the most critical mechanical properties of nanofibers. Tensile strength is usually tested using a universal testing machine (UTM), also known as tensile testing machine or tensile strength instrument, following appropriate testing standards such as EN ISO 13934-1:1999 for the strip method [131]. A UTM applies a controlled force to a nanofiber sample and measures the force and the corresponding deformation. Nanofibers are typically collected in the form of mats or films, which are cut into small strips and mounted between the grips of the UTM. The force is applied until the material ruptures, and the tensile strength is calculated based on the force per unit area. Unlike testing of bulk materials, tensile testing of electrospun materials with the characteristics of a thin, porous, and soft fibrous membrane can be tricky in terms of handling and thickness measurements used for stress calculations [132]. Preparation of specimens requires attention to prevent damage, pretension, or slipping from grips during testing. Maccaferri et al. [133] demonstrated the use of a paper frame to hold the specimen in place for better handling and positioning on the machine, as shown in Figure 9.

Tensile properties measured by UTM include ultimate tensile strength, Young’s modulus, and elongation at break [134]. Ultimate tensile strength is the maximum stress the specimen can withstand before rupturing. Young's modulus (or elastic modulus) is a measurement of elasticity, which describes the stiffness of nanofibers and is calculated as the ratio of stress to strain in the elastic deformation region, which is the slope of the initial linear region of the stress–strain curve. Elongation at break measures the extent to which the nanofiber can stretch before it breaks, expressed as a percentage of its original length. This property is important for applications that require flexibility, such as in wound dressings. Toughness is a measure of the energy a material can absorb and plastically deform before failure. It can be derived from the area under the stress–strain curve obtained during tensile testing.

The tensile strength values can vary depending on the composition, polymer concentration, and electrospinning parameters. Different studies in the literature have shown inconsistent effects of chitosan-to-PVA ratio on tensile properties of the material. Koosha et al. [59] observed a reduction in ultimate tensile strength, Young’s modulus, elongation at break, and toughness with the incorporation of chitosan as opposed to pure PVA membranes. As the chitosan content increases, the tensile strength decreases because of the less favorable mechanical properties of chitosan compared to PVA. For the chitosan/PVA membrane, the stiffer nature of chitosan tends to reduce flexibility, while toughness tends to decrease because of the brittleness of chitosan. In support of these observations, the effects of different chitosan-to-PVA ratios in cross-linked chitosan/PVA nanofibers investigated by Zhou et al. [135] shows improvement in tensile strength, elongation at break, and Young’s modulus with increasing PVA content from 10% (w/w) to 50% (w/w). Choo et al. [136] measured the tensile strength and elongation at break of pure chitosan, pure PVA, and different weight ratios between chitosan and PVA. Interestingly, although the elongation at break increased with higher PVA content, tensile strength was reduced, which could be caused by the formation of a more single-ordered phase of PVA in the structure. Bonilla et al. [137] also observed the largest elongation for pure PVA, whereas the tensile strength and Young’s modulus were optimal at a specific chitosan-to-PVA ratio of 20:80.

In contrast, Tan et al. [120] reported improved tensile properties with a larger elongation at break for chitosan/PVA nanofibers as compared to pure PVA nanofibers, said to be due to hydrogen bond formation between chitosan and PVA. Olvera Bernal et al. [58] studied different concentrations of chitosan between 2.5% and 4% (w/w) in the chitosan/PVA membrane, while keeping the PVA concentration constant at 5% (w/w). The Young’s modulus was found to increase with increasing chitosan concentration, but only up to 3.5% (w/w); further increasing the concentration resulted in a significant reduction in elasticity. The elongation at break was also highest at a chitosan concentration of 3.5% (w/w). These results indicate that the mechanical properties of chitosan/PVA nanofibers depend on the chitosan and PVA content and should be optimized to obtain nanofiber membranes with good mechanical strength. Table 5 shows the measured mean values of different tensile properties for several material combinations tested.

**Table 5:** Measured tensile properties of chitosan/PVA nanofibers from the literature.

Material	Ultimate tensile strength (MPa)	Young’s modulus (MPa)	Elongation at break (%)	Toughness (MJ/m^3^)	Reference

chitosan/PVA	2.82–6.42	299.7–648.45	3.12–5.92	—	[58]
chitosan/PVA	3.34	0.282	6.7	6.02	[134]
chitosan/PVA + graphene nanoplatelets	4.35–11	0.8–2.82	8.02–22	45–130.9	
chitosan/PVA	0.17	—	—	—	[16]
chitosan/PVA/PVDF	0.65	—	—	—	
chitosan/PVA	6.6	153.5	10.1	—	[138]
chitosan/PVA	2.2	36.5	30.8	—	[139]
chitosan/PVA + glutaraldehyde	2.9–4.6	69.0–198.1	11.5–25.1	—	
chitosan	27.4	—	26.7	—	[136]
chitosan/PVA	18.8–21.1	—	45.4–127.5	—	
PVA	13.4	—	220.4	—	
chitosan/PVA	5.26	190	4.5	0.153	[59]
PVA	7.4	280	50.2	2.70	
chitosan	20	533	6	—	[137]
chitosan/PVA	24–43	172–1390	32–173	—	
PVA	23	263	265	—	
chitosan/PVA	2.78–5.55	91.9–167.7	10.58–36.56	—	[135]

#### Viscoelastic properties

Viscoelasticity is another property relevant to nanofibers, which combines both elastic and viscous responses to deformation. Viscoelastic properties, including storage modulus and loss modulus, can be tested using dynamic mechanical analysis (DMA) or dynamic mechanical thermal analysis (DMTA). This technique involves applying a sinusoidal stress or strain to the material and measuring the resulting strain or stress, allowing for the characterization of time-dependent deformation of the material. The parameters varied in the tests include oscillation amplitude and frequency, while DMTA tests include temperature as the additional parameter [140].

The presence of chitosan in PVA membranes is expected to increase the storage modulus because of the higher stiffness of chitosan chains compared to PVA chains, as reported from DMA tests by Parparita and coworkers [141]. Koosha et al. [59] measured the dynamic mechanical and thermal properties of the PVA and chitosan/PVA nanofiber membranes using DMTA and observed an opposite trend of higher storage modulus for the PVA membrane compared to the chitosan/PVA membrane at temperatures lower than 80 °C. The authors explained several possibilities that resulted in this contradictory observation, including reduced crystallinity as evidenced by the X-ray diffraction results and the presence of bound solvent detected through differential scanning calorimetry, which acts as plasticizer in the chitosan/PVA membrane and reduces the storage modulus. Viana et al. [138] conducted DMA of cross-linked and not cross-linked chitosan/PVA nanofibers to investigate the variation in loss factor with temperature, which is obtained from the ratio of loss modulus to storage modulus. The increase in loss factor observed after cross-linking for 48 h indicates high cross-linking density and smaller distance between molecular chains, which improves energy dissipation. Saeedi et al. [142] conducted DMTA tests and found that storage modulus and loss modulus of chitosan/PVA improved with the addition of graphene oxide. An improvement of loss modulus indicates resistance of polymer chains against viscoelastic deformation and sliding.

#### Morphological properties and structure

Mechanical properties can often be correlated with the morphology and structure of the nanofibers [143]. The use of optical microscopes in obtaining nanofiber images is quite rare because of the relatively low resolution, but it is not impossible. This was demonstrated by Olvera Bernal et al. [58] who employed differentially interferential contrasting to accentuate the color contrast for the fiber diameter measurements of electrospun chitosan/PVA. Imaging techniques based on the principles of electron beams such as scanning electron microscopy (SEM) and field-emission scanning electron microscopy (FESEM) are more commonly used to analyze the fiber diameter, distribution, and overall surface morphology [144]. Microscopic images obtained from these techniques help to identify defects such as beading or non-uniformity in fibers, which can adversely affect mechanical performance. Besides SEM and FESEM, transmission electron microscopy (TEM) has the additional ability to visualize fiber cross sections and can be employed to examine core–shell, encapsulated, and particle-incorporated fiber structures [112,145–146]. Atomic force microscopy has been used to investigate morphology as well as the nanomechanical properties of individual fibers, including magnetization, friction, and mechanical strength [147].

These imaging techniques are critical for linking the mechanical behavior to the microstructure of the fibers. Based on fiber diameter measurements from SEM, Hartatiek et al. [148] observed an inversely proportional correlation between fiber diameter and the ultimate tensile strength of the chitosan/PVA/collagen nanofibers, with the smallest average diameter of 154 nm resulting in the highest tensile strength of 5.6 MPa due to higher connectivity and stronger bonding between the finer fibers. Kuo et al. [112] fabricated core–shell fibers with chitosan as the shell layer and PVA as the core layer and tested the effects of different chitosan contents on the mechanical properties. The tensile strength improved with lower chitosan concentration because of improved coverage of the chitosan shell layer as observed from TEM images of the fiber.

The different methods used in the measurement of chitosan/PVA nanofiber properties are summarized in Table 6. These methods allow for comprehensive characterization of the mechanical properties of electrospun nanofibers, aiding in the optimization of their performance for various applications.

**Table 6:** Methods used in the measurement of morphological and mechanical properties of chitosan/PVA nanofibers.

Properties	Characterization method	References

tensile properties, that is, ultimate tensile strength, Young’s modulus, elongation at break, and toughness	universal testing machine	[16,62,112,125,139,149]
	dynamic mechanical analysis	[118]
viscoelastic properties, storage modulus, and loss modulus	dynamic mechanical thermal analysis	[149]
	dynamic mechanical analysis	[139,150]
fiber morphology and diameter	scanning electron microscopy	[103,117,139,148,151]
	field-emission scanning electron microscopy	[16,119]
	transmission electron microscopy	[112,145–146,152]
	atomic force microscopy	[145,153–154]
	optical microscopy with differentially interferential contrasting technique	[58]

### Strategies for mechanical improvement

There are various methods to improve the mechanical properties of electrospun nanofibers. These methods can generally be divided into two categories, namely, material modifications and structural modifications. Material modifications include mixing with other polymers, plasticizers, and additives [155], as well as surface modification [156] and post-treatment processes such as cross-linking and heat treatment [157]. Structural modifications encompass fiber alignment, multilayer structures, and core–shell structures [158].

#### Structural modifications

Structural modifications of electrospun nanofibers, including modifications on fiber orientation, layering of nanofibers, and core–shell structures can alter the mechanical properties of the material. For chitosan/PVA nanofibers, the addition of PVA to chitosan or vice versa, either through blending or by layering can improve mechanical properties of the material. Mohd Salleh et al. [16] prepared bilayer chitosan/PVA nanofibers, which were found to improve tensile strength through strong physical interactions between the two layers. Rafieian et al. [109] incorporated PVA nanofibers onto chitosan/aloe vera films to improve the mechanical, physical, and biological properties of the films for wound dressing applications. Kim et al. [159] compared the mechanical properties of pure PVA, blended chitosan/PVA, and PVA core–chitosan sheath nanofiber membranes; they found that the addition of chitosan to PVA reduced the tensile strength but increased the Young’s modulus of the membranes. However, the effects of different chitosan-to-PVA ratios on the mechanical properties reported in the literature varies, as discussed above (section “Characterization of mechanical properties”, “Tensile properties”). For core–shell nanofiber structures, the mechanical properties can be enhanced by selecting a stronger material as the core layer [112].

Several methods were also employed to modify the surface properties of electrospun nanofibers, which include blending of functional agents in the polymer solution, wet chemical methods, surface graft polymerization, and plasma treatment [160]. Low-temperature plasma offers advantages over other techniques in terms of lower energy consumption, faster processing, and minimal solvent use [161]. By appropriately selecting the plasma source and controlling discharge conditions such as voltage, pressure, and gas flow rate, various functional groups can be introduced on the fiber surface, enabling tailored modifications that enhance polymer biocompatibility. Punamshree et al. [162] performed surface modification of chitosan/PVA nanofibers using dielectric barrier discharge (DBD) plasma and reported enhanced wettability, mechanical properties, and biocompatibility of the fibers. The argon and oxygen plasma treatments led to significant cross-linking of the fibers, which improved tensile strength and Young’s modulus, and an increase in surface roughness, which reduced the water contact angle, without altering the bulk properties of the material. Similar observations of improved mechanical strength of plasma-treated chitosan/PVA/hydroxyapatite were reported using DBD plasma air, which is considered more cost-effective as no vacuum pump is required in the process [148]. Incorporation of nanofiller into the nanofiber matrix, such as in the halloysite nanotube-reinforced chitosan/PVA nanofibers prepared by Koosha et al. [163], can also improve mechanical properties. Rosli et al. [164] functionalized an ionic liquid, 1-allyl-3-methylimidazolium chloride into cross-linked electrospun chitosan/PVA nanofibers to improve water resistance and adsorption properties. The mechanical properties, however, were not reported. Coating of nanofiber structures can also provide improvement of the mechanical properties. Kang et al. [110] coated electrospun PVA fibers with chitosan solution and reported improved tensile properties due to interfiber bonds created by the chitosan coating.

#### Material modifications

In recent research, electrospun chitosan/PVA nanofibers have been mixed with silk fibers, resulting in significantly improved mechanical properties [125]. The solutions of each component were prepared, combined in specific volume ratios, and then electrospun to form nanofibers from both the pure PVA and chitosan/PVA mixture, as well as the silk solution. The incorporation of co-electrospun silk into the chitosan/PVA fibrous mat significantly enhanced its mechanical strength and Young's modulus, achieving values more than twice of those of PVA or chitosan/PVA fibrous mats. This improvement was attributed to the chemical interactions between the co-electrospun silk and the blended chitosan/PVA [83,165]. The PVA fiber mat failed at approximately 100% elongation, while the breaking strain increased to about 220% with the addition of chitosan and silk in the composite fibrous mat.

Another technique for improving mechanical properties is the addition of fillers, such as nanomaterials. Reinforced polymeric nanocomposites have been produced through the substantial utilization of nanoparticles. The development of fibrous nanocomposites or bio-nanocomposites, where the matrix and/or fillers are biomaterials, has been advanced in recent years by introducing nanoscale materials into electrospun fibers in the form of particles, fibers, and whiskers [166–168]. Chitosan/PVA/nanoclay composite nanofibers were successfully prepared by electrospinning. Chitosan and PVA solutions were prepared and mixed in a 30:70 volume ratio. Montmorillonite nanoclay was then dispersed in acetic acid and mixed with the chitosan/PVA solution. The solutions were electrospun immediately after preparation, producing bead-free nanofibrous mats with enhanced mechanical properties [149]. Young’s modulus, ultimate tensile strength, elongation at break, and toughness of electrospun nanofibers with nanoclay were significantly increased compared to electrospun fibers without nanoclay. This improvement is due to the dispersion of nanoparticles within the polymer matrices, which plays an important role in enhancing the physical and mechanical properties of electrospun nanofiber [169].

The purpose of surface modification is to increase the number of adsorption sites on the surface, improve mechanical properties, enhance hydrophilicity and wettability, and stabilize nanofibers in aqueous solutions [156]. Numerous techniques have been developed to modify the surface properties of electrospun nanofibers, including surface graft polymerization, wet chemical procedures, low-temperature plasma treatment, and co-electrospinning with surface-active chemicals and polymers [160]. Among these methods, low-temperature plasma treatment is particularly popular because it can modify the surface properties of the nanofibers without affecting their bulk properties [170–171]. This technology is advantageous in terms of energy consumption, processing time, and solvent use, and it is also non-hazardous to the environment [161].

In a study on the surface modification of electrospun chitosan/PVA nanofibers, PVA was dissolved in distilled water and mixed with chitosan dissolved in acetic acid in a 1:4 ratio. The solution was electrospun into nanofibers, and plasma treatment was carried out using DBD plasma with O_2_ gas. The treated electrospun nanofiber exhibited a 15.6% increase in tensile strength and a 37.3% increase in modulus. This phenomenon can be explained by the formation of strong hydrogen bonds in the chitosan/PVA nanofiber matrix induced by the O_2_ plasma, leading to a high cross-link density in the nanofibers and resulting in substantial load transfer to the nanofiber matrix [172]. Additionally, the O_2_ plasma treatment assists in the formation of chemical (intermolecular and intramolecular hydrogen) bonds, which also lead to a highly cross-linked chitosan/PVA nanofiber matrix [162]. Table 7 summarizes the enhancement of material modifications on the mechanical properties of electrospun nanofiber.

**Table 7:** Effect of material modifications on the mechanical properties of electrospun nanofibers.

Material modification	Sample	Elastic modulus (MPa)		Tensile strength (MPa)	

		before modification	after modification	before modification	after modification

mixing with other polymers	chitosan/PVA and silk electrospun fibers seeded with differentiated keratinocytes	0.4	2.0	0.65	2.2
addition of fillers	nanoclay-reinforced electrospun chitosan/PVA nanocomposite nanofibers	190	858	5.26	15.45
surface modification using dielectric barrier discharge oxygen plasma	surface modification of electrospun PVA/chitosan nanofibers by dielectric barrier discharge plasma	69.76	111.3	20.5	24.3

Another method to improve the mechanical properties of polymeric elastomers is cross-linking. Cross-linking is a crucial process for enhancing the mechanical elasticity, toughness, and stability of polymers [173]. It can be categorized into two types, that is, chemical and physical. Chemical cross-linking involves the formation of covalent bonds between polymer chains, resulting in structures that are stable under deformation, exhibiting high stiffness and mechanical elasticity. This process is often facilitated by agents such as glutaraldehyde [174], epichlorohydrin, genipin, and citric acid [164].

**Figure 10:**
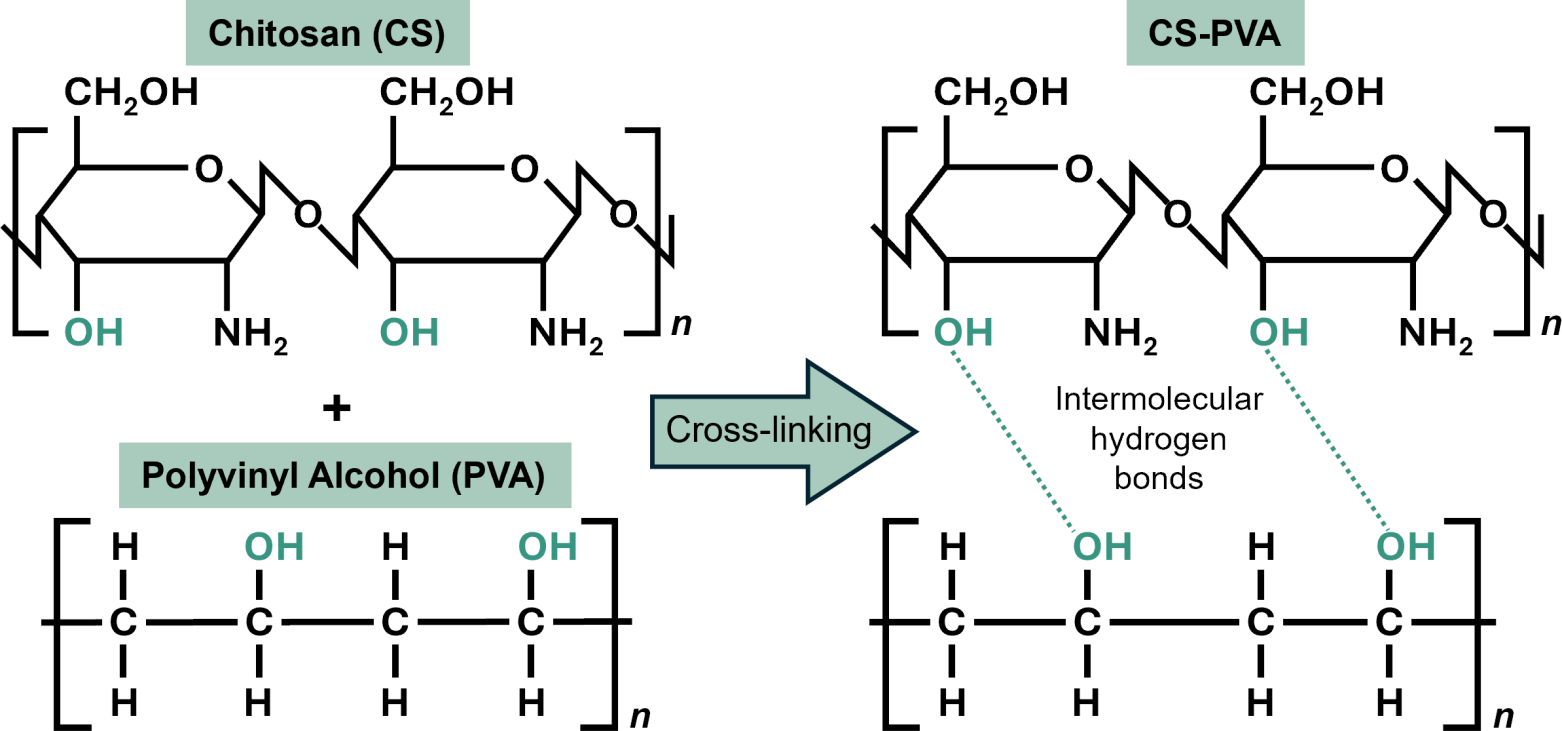
The mechanisms of cross-linking for chitosan/PVA composites.

Physical cross-linking involves non-covalent bonds, such as hydrogen bonds, ionic interactions, coordinate bonds, and dynamic covalent bonds [175]. These bonds are generally weaker than chemical cross-links and can break under deformation. However, physical cross-linking is considered more environmentally friendly compared to chemical cross-linking [176–177], which often employs toxic chemicals. An example of physical cross-linking is heat treatment, which has been extensively used to cross-link PVA [178]. Figure 10 illustrates the mechanisms of cross-linking in chitosan/PVA composites.

In one study, electrospun chitosan/PVA nanofibers were loaded with ampicillin sodium for burst drug release, and glutaraldehyde was applied as chemical cross-link to the nanofibers [139]. This treatment produced fine morphology and enhanced mechanical properties. The Young’s modulus improved from 36.5 to 198.1 MPa, and the tensile strength increased from 2.2 to 4.6 MPa. This significant improvement in Young’s modulus and tensile strength was attributed to the formation of a cross-linked network within the PVA/CS composite nanofibers, which restricted the movement of molecular chains [179].

Another study on electrospun PVA nanofibers used heat treatment as a physical cross-linking method and found that, at 85 °C, the Young’s modulus increased from 0.110 to 0.137 MPa, and the tensile yield stress improved from 3.63 to 9.63 MPa [180]. Heat treatment induces intramolecular cross-linking within the PVA chains and causes dehydration of the PVA sample. This process leads to a decrease in the number of hydroxy groups present on the PVA chain, resulting in the release of water molecules. Simultaneously, the remaining hydroxy groups facilitate the formation of intramolecular hydrogen bonds. Additionally, intermolecular hydrogen bonds can form between PVA chains, increasing the crystallinity and enhancing the mechanical properties [181–182]. Table 8 shows the cross-linking effect on mechanical properties of electrospun nanofibers.

**Table 8:** Effects of cross-linking on the mechanical properties of electrospun nanofibers.

Types of cross-linking	Sample	Elastic modulus (MPa)		Tensile strength (MPa)		Reference

		before cross-linking	after cross-linking	before cross-linking	after cross-linking	

chemical cross-linking	electrospun chitosan/PVA nanofibers cross-linked with glutaraldehyde	36.5	198.1	2.2	4.6	[139]
physical cross-linking	PVA nanofibers with heat treatment (85 °C)	0.110	0.137	3.6	9.6	[180]

### Applications

One of the unique features of core–shell chitosan-based electrospun nanofibers is their capability for efficient sustained release. These nanofibers also exhibit better stability, which are vital properties in drug delivery systems [183–184]. These superior properties have gained recent attention for enhancing the functional performance of chitosan-based nanofibers. Core–shell chitosan nanofibers have been utilized in various specialized applications, such as scaffolds in tissue engineering [185], controlling drug release mechanisms through the manipulation of core and shell compositions [186], and protecting sensitive biomolecules in food packaging [187]. The combination of core–shell chitosan-based nanofibers with different materials has a significant impact on protecting sensitive drugs through the shell layer, reducing burst release, and controlling the release of both hydrophilic and hydrophobic drugs [188–189]. In recent research, chitosan/PVA nanofibrous scaffolds loaded with active components from *Satureja mutica* and *Oliveria decumbens* were successfully electrospun to produce a core–shell structure. This modification was found to enhance the mechanical properties compared to previous chitosan/PVA scaffolds using other approaches. Additionally, the incorporation of these active components improved the antimicrobial activity of the nanofibers against a wide range of bacteria [190].

In tissue engineering applications, aligned fibers are particularly effective as they better mimic the inductive environment, such as that of human tendon stem/progenitor cells, compared to random fibers [191]. Aligned fibers also guide human mesenchymal stem cells toward cardiomyogenesis and enhance myoblast differentiation [192–193]. Research has demonstrated that aligned nanofibers can accelerate the recovery of highly organized structures, such as nerve cells, tendons, and ligaments. Additionally, aligned nanofibers provide topographic guidance to cells, promoting cell adhesion, proliferation, and migration. Most importantly, aligned fibers are a prominent type of nanofiber that closely mimics the fibers in the ECM of native tissue [194]. Radially aligned nanofiber patches of chitosan/PVA with thyme essential oil were fabricated for the application of repairing chronic tympanic membrane perforations [195].

In drug release applications, multilayered electrospun nanofibrous mats can enhance both the physical properties and the release period of active compounds [196–197]. Additionally, chitosan exhibits unique properties such as biodegradability, biocompatibility, and antibacterial activity, which are considered effective in promoting wound healing. The presence of chemical structures like glycosaminoglycans in chitosan mimics the ECM and provides a hemostatic effect, thereby accelerating the healing process [198–201]. In one study, multilayered nanofibrous patches consisting of PCL nanofibers, chitosan/PVA nanofibers, and chamomile were successfully prepared. The results demonstrated that these multilayered nanofibrous mats exhibited comparable mechanical and swelling properties, along with excellent antibacterial efficiency, when compared to commercial wound dressings [202].

### Challenges and future perspectives

Despite new technologies being discovered every day, the current mechanical performance of electrospun nanofibers remains limited. For biomedical materials, single modification methods are still predominantly used, even though combining two methods is more efficient. For instance, the combination of electrospinning with stretching and annealing treatments has been shown to produce ultrafine nanofibers [25].

Another issue with electrospun scaffolds is balancing their function of mimicking the nanomechanical and nanostructural features of the ECM with their ability to withstand load-bearing applications, given their high porosity. Additionally, conventional electrospun fibers cannot withstand 150 MPa, which is a requirement for load-bearing applications. Thus, a more robust method is needed to address this limitation. This method should not only support cell spreading, cell adhesion, and ECM synthesis but also withstand external forces to maintain their integrity and function without collapsing [203].

A further critical issue are environmental and user safety concerns. In most chemical cross-linking processes, bifunctional agents such as glutaraldehyde, diisocyanates, and carbodiimides are used. Despite their effectiveness in enhancing the mechanical properties of electrospun nanofibers, these cross-linking agents exhibit high toxicity, not only to cells and biological systems but also to the environment [204]. Therefore, the development of green cross-linking methods should be pursued in the future [205].

## Conclusion

Electrospinning offers a versatile and cost-effective method for fabricating polymeric nanofibers, with chitosan/PVA electrospun nanofibers showing promise in water treatment, biomedical uses, and wound healing. However, their mechanical properties are a significant challenge because of high porosity, random fiber orientation, and weak interactions at fiber cross-points.

Our review highlights various strategies to enhance the mechanical properties of chitosan/PVA electrospun nanofibers. Material modifications, such as blending with other polymers like silk, incorporating nanomaterials, and surface treatments like plasma exposure, significantly improve tensile strength, Young’s modulus, and elongation at break. Structural modifications, including fiber alignment, multilayer, and core–shell structures, also enhance mechanical performance. Cross-linking, both chemical and physical, creates more robust and stable nanofiber networks.

Despite these advancements, challenges remain in achieving the desired mechanical strength for specific applications, especially load-bearing scenarios. Future research should focus on optimizing modification techniques to achieve the best mechanical performance while maintaining the benefits of electrospun nanofibers. Additionally, addressing environmental and user safety concerns associated with chemical cross-linking agents will be crucial.

Through continuous innovation and interdisciplinary collaboration, the potential of chitosan/PVA electrospun nanofibers can be fully realized, paving the way for their broader use in advanced technological and medical applications.

## Data Availability

Data sharing is not applicable as no new data was generated or analyzed in this study.
